# Massively Parallel Interrogation of Aptamer Sequence, Structure and Function

**DOI:** 10.1371/journal.pone.0002720

**Published:** 2008-07-16

**Authors:** Nicholas O. Fischer, Jeffrey B.-H. Tok, Theodore M. Tarasow

**Affiliations:** 1 Chemistry, Materials, Earth and Life Sciences Directorate, Lawrence Livermore National Laboratory, Livermore, California, United States of America; 2 Tethys Bioscience, Inc., Emeryville, California, United States of America; University of Helsinki, Finland

## Abstract

**Background:**

Optimization of high affinity reagents is a significant bottleneck in medicine and the life sciences. The ability to synthetically create thousands of permutations of a lead high-affinity reagent and survey the properties of individual permutations in parallel could potentially relieve this bottleneck. Aptamers are single stranded oligonucleotides affinity reagents isolated by *in vitro* selection processes and as a class have been shown to bind a wide variety of target molecules.

**Methodology/Principal Findings:**

High density DNA microarray technology was used to synthesize, in situ, arrays of approximately 3,900 aptamer sequence permutations in triplicate. These sequences were interrogated on-chip for their ability to bind the fluorescently-labeled cognate target, immunoglobulin E, resulting in the parallel execution of thousands of experiments. Fluorescence intensity at each array feature was well resolved and shown to be a function of the sequence present. The data demonstrated high intra- and inter-chip correlation between the same features as well as among the sequence triplicates within a single array. Consistent with aptamer mediated IgE binding, fluorescence intensity correlated strongly with specific aptamer sequences and the concentration of IgE applied to the array.

**Conclusion and Significance:**

The massively parallel sequence-function analyses provided by this approach confirmed the importance of a consensus sequence found in all 21 of the original IgE aptamer sequences and support a common stem:loop structure as being the secondary structure underlying IgE binding. The microarray application, data and results presented illustrate an efficient, high information content approach to optimizing aptamer function. It also provides a foundation from which to better understand and manipulate this important class of high affinity biomolecules.

## Introduction

The ability to generate high affinity binding molecules on demand has and will continue to have profound impact on medicine and the life sciences. While methods for the discovery of affinity reagents directed against a particular target take many forms and have been refined over many years, the rate-limiting step to applying these reagents is often the optimization of properties such as affinity, specificity, size and impact of any chemical modifications. The ability to synthetically create thousands of permutations of the original affinity reagent and survey the properties of individual permutations in parallel could potentially relieve this bottleneck. The opportunity to obtain binding data for thousands of variations would thus not only streamline application development but also provide valuable data about what permutations lead to observed properties. Extensive data sets that connect measurable, functional properties with varied composition and predicted topology of the affinity reagent may ultimately lead to a better understanding of structure and function.

Aptamers are single stranded oligonucleotides that bind with high affinity to a given target. They are derived using the Systematic Evolution of Ligands by Exponential Enrichment (SELEX), an *in vitro* selection process that isolates specific sequences (aptamers) from extremely large random oligonucleotide sequence pools (libraries) [1], [2]. The SELEX process has been used to isolate single stranded DNA (ssDNA) aptamers for a wide array of targets including small molecules [3], peptides [4], proteins [5] and even whole cells [6]. Aptamers typically bind their protein targets with dissociation constants in the picomolar to nanomolar range [7] and can discriminate against, for example, closely related proteins [8]. In addition to a proven and scalable discovery process and desirable biophysical properties, DNA aptamers are an attractive biomolecular platform for pharmaceutical, diagnostic and life science tool applications because they can be chemically synthesized and modified, can be routinely denatured and renatured and are relatively stable under a variety of condition [5]. However, as with other molecular platforms, aptamers that are isolated from a SELEX typically require optimization for a given application.

As only a portion of the full length aptamer sequence obtained by SELEX is usually required for analyte binding [9], a key, practical driver in aptamer optimization is to maintain a high level of function while decreasing the oligonucleotide sequence to its minimal functional length. Hence, aptamer optimization typically involves creating a series of truncates and measuring activity to identify the minimal sequence responsible for target binding [10]. This iterative process can typically require months to survey and optimize just a handful of aptamers. A method that could simultaneously and discretely survey thousands of individual aptamer sequence permutations would greatly accelerate this process. In addition, the coupling of large amounts of sequence and binding data could lead to a better understanding of aptamer structure and function relationships, which in turn may lead to an ability to optimize aptamer function or even design aptamers de novo. In this report we describe the synthesis and characterization of thousands of aptamer sequences in parallel using microarray technology. The full-length sequences and 175 truncated versions of each of 21 previously described, IgE binding aptamers were studied in parallel producing essentially 3,900 experiments in triplicate and yielding thousands of data points relating aptamer sequence to function.

Microarray technology has progressed to the point where high density DNA arrays with customized content can be rapidly prepared on demand. The ability to create these highly parallel, high information content experimental platforms has been enabled by various “maskless” methods of *in situ* oligonucleotide synthesis array production including electrode array electrochemical methods (www.combimatrix.com), Digital Micromirror optical methods (www.nimblegen.com) and inkjet printing methods (www.agilent.com). The latter, developed by Agilent Technologies, allows for the synthesis of oligonucleotides up to 200 nucleotides in length; relatively long compared to other technologies and well suited for synthesizing and studying aptamers. Prior reports of aptamer arrays utilized spotting technology wherein oligonucleotide sequences are synthesized off-chip using standard synthetic processes and then deposited/immobilized onto the chip [11]–[14]. The application of in-situ synthesized, high density DNA microarray technology has the potential to increase by orders of magnitude the number of sequences surveyed and data produced per array and provide a wealth of information to accelerate our understanding of aptamer structure and function. As an example, this approach has recently been applied to measuring the effects on function of single- and multi-base mutations of a single, truncated aptamer [15]. As a further demonstration of this potential and the first high density microarray containing full length aptamers, we chose to study previously reported immunoglobulin E (IgE) DNA aptamers [16] using Agilent's high density microarrays. These IgE aptamers were chosen because more than 25 unique sequences have been reported, all of which share a common 21 nucleotide sequence. In addition, truncation studies of a single IgE aptamer sequence, D-17, identified a putative stem-loop structure as being responsible for target binding. These results provided a useful foundation from which to use microarrays to study thousands of these IgE-binding aptamers and truncates for sequence, function and structure relationships.

## Results

**Figure 1:**
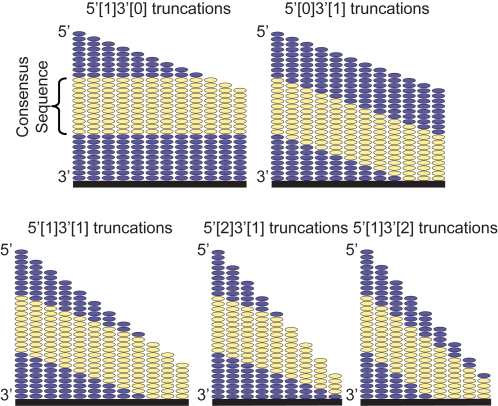
Schematic depicting the effects of truncation on consensus sequence position relative to array surface. Single 5′ truncations, 5′[1]3′[0], expose the consensus sequence without affecting the relative position of the consensus sequence. Conversely, single 3′ truncations, 5′[0]3′[1], systematically decrease the distance between the consensus sequence and the array surface while leaving non-binding 5′ regions intact. Truncations from both termini (5′[1]3′[1], 5′[2]3′[1], and 5′[1]3′[2]) result in a combination of both effects.

**Figure 2:**
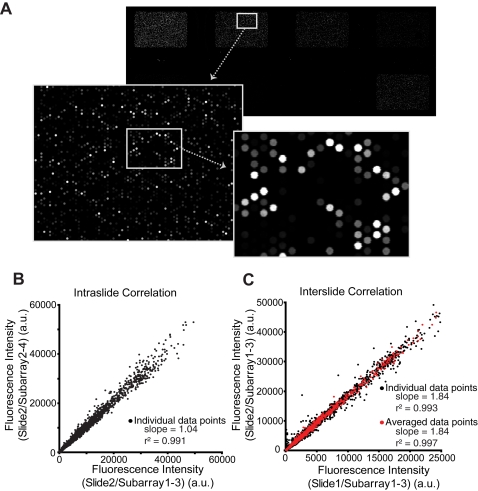
Aptamer array features are uniform, discrete, and highly reproducible. (A) Representative images of an aptamer microarray incubated with labeled IgE. The eight identical subarrays were individually yet simultaneously interrogated with decreasing concentrations of IgE (100 nM to 0.1 nM). Insets (30 nM IgE) demonstrate discrete features of uniform size and intensity. (B,C) Subarrays incubated with 10 nM IgE from two separate experiments were used to analyze intra- and inter-slide data correlation (B and C, respectively). Both correlation analyses demonstrated a high degree of reproducibility, with r^2^ values greater than 0.990. The slope of the correlation trend is indicative of relative fluorescence intensities. Slide 2 was washed less stringently than slide 1, hence the higher fluorescence intensities of its features are reflected in a slope of 1.84 (vs. 1.04 in the intraslide comparison where all wash conditions were constant). Averaging of triplicate data points (c, red circles) effectively eliminated outliers seen in the plot of individual data points (black circles). Average percent coefficient of variability (%CV) values for intra- and interslide subarray comparisons were determined to be 5.76% and 6.86%, respectively (see Figure S2).

Aptamer arrays were designed to simultaneously interrogate several different truncation series of 21 distinct IgE-binding aptamers in a single experiment. The microarrays featured 8 identical subarrays, each consisting of *ca.* 3,900 unique test sequences, each of which was synthesized in triplicate. Five sets of serial truncations were incorporated into the array design: single 5′ only (5′[1]3′[0]), single 3′ only (5′[0]3′[1]), single 5′ with single 3′ (5′[1]3′[1]), single 5′ with double 3′ (5′[1]3′[2]), and double 5′ with single 3′ (5′[2]3′[1]) (**Figure 1**). A PDGF-binding aptamer and its truncations were included as negative controls [8] (see **Table S1** for complete list of full length aptamers and sequences). IgE labeled with DyLight 549 was used to probe the binding function of the arrayed oligonucleotides. Arrays were typically incubated with labeled IgE for two hours and washed before IgE binding at each feature was determined by measuring fluorescence intensity using a microarray scanner (Agilent Technologies). Casein was used as a blocking agent to help minimize non-specific background fluorescence. A representative image of an array incubated with IgE is shown in **Figure 2A**. The array scan demonstrates that individual features were of uniform size and intensity. Furthermore, features were well resolved from one another with minimal background fluorescence and significant dynamic range of on-feature fluorescence intensity. In addition, little fluorescence was observed in the control features presenting non-IgE binding oligonucleotides. Blocking and incubation conditions were optimized for signal to noise, however, it was generally observed that good results could be obtained under all of the conditions explored (**Figure S1**). The reproducibility of the array measurements was examined by comparing the results between subarrays on the same chip and between subarrays from different chips (all incubated with 10 nM IgE). As can be seen in **Figures 2B** and **2C**, the correlations in both cases were strong, demonstrating that the same relative intensities were achieved chip-wide between experiments (also see **Figure S2**). Comparison of the individual feature level data (**Figure 2C**), where outliers are clearly obvious, with the improved correlation of the averaged data underscores the value of being able to interrogate sequences in triplicate in the same experiment thus averaging out what appear to be spurious individual results.

**Figure 3:**
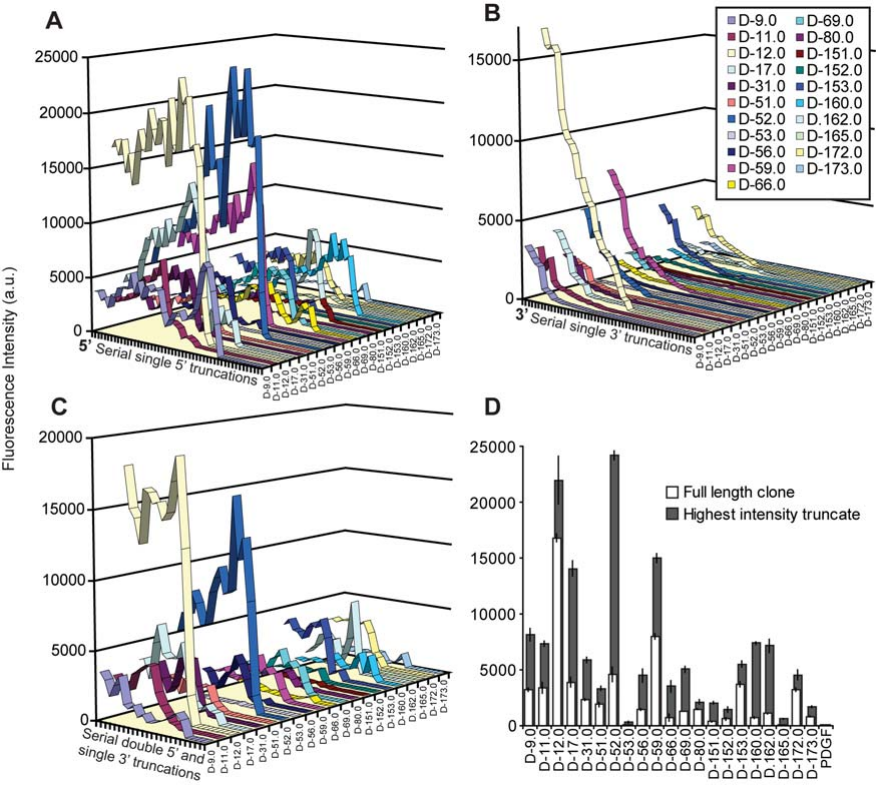
Examining the relationship between sequence and function. Complete graphical representation of (A) 5′[1]3′[0] truncations, (B) 5′[0]3′[1] truncations, and (C) 5′[2]3′[1] truncations. Aptamer arrays were incubated with 10 nM labeled IgE. Each point represents a single truncate (average of triplicate measurements), sequentially ordered from left to right according to the full length clone sequence. (D) Summary of full length and highest fluorescence intensity truncate for each clone. All clones exhibit truncates with higher intensities than the full length sequences. The small error (error bars are 1 s.d. of triplicate data points) demonstrates high degree of reproducibility throughout the array.

For any given experiment, the fluorescence intensity at each feature was mapped to the corresponding sequence for sequence – function analysis. **Figure 3** shows the cumulative data for three truncation studies across all of the 21 sequence families, plotted sequentially according to the full-length sequence. It is important to note that the level of IgE binding as measured by fluorescence intensity was measured in triplicate for each sequence and that each graph in **Figures 3A–C** represents more than eight hundred data points. Several general trends are evident across the different truncates (**Figure 3** **)**. First, most of the 5′ truncates, whether 5′ truncates only or combinations with 3′ truncation, demonstrated a general increase in fluorescence signal out to a given point where there was a precipitous drop to no IgE binding as measured by a lack of fluorescence. The data from the 3′ truncation series were noticeably different from the 5′ series. In general, the 3′ truncations showed a slow degradation of fluorescence intensity until a complete loss of IgE binding occurred. **Figure 3D** summarizes the fluorescence intensities of the full length and the highest fluorescence intensity truncated sequences for all clones. Neither a full-length negative control aptamer (anti-PDGF) nor its truncates exhibited any detectable binding to IgE (**Figure 3D**).

To explore fluorescence intensity as a function of target protein concentration, arrays were incubated with IgE ranging from 0.1 to 300 nM. Increased protein binding was observed across the entire range of IgE concentrations with a slight indication of approaching saturation for some of the sequences at the highest IgE concentration (**Figure S3**). These data indicate that, even when displayed on the array surface, the K_d_s of these aptamers are >100 nM. In contrast, previous studies measured the K_d_ of the full length sequence from clone D-17.0 to be 9 nM in solution [16]. However, significant IgE binding was observed at concentrations as low as 0.1 nM, demonstrating the sensitivity of the measurements and underscoring the good signal to noise ratios obtained with this measurement platform.

## Discussion

### Sequence – function relationships

**Figure 4:**
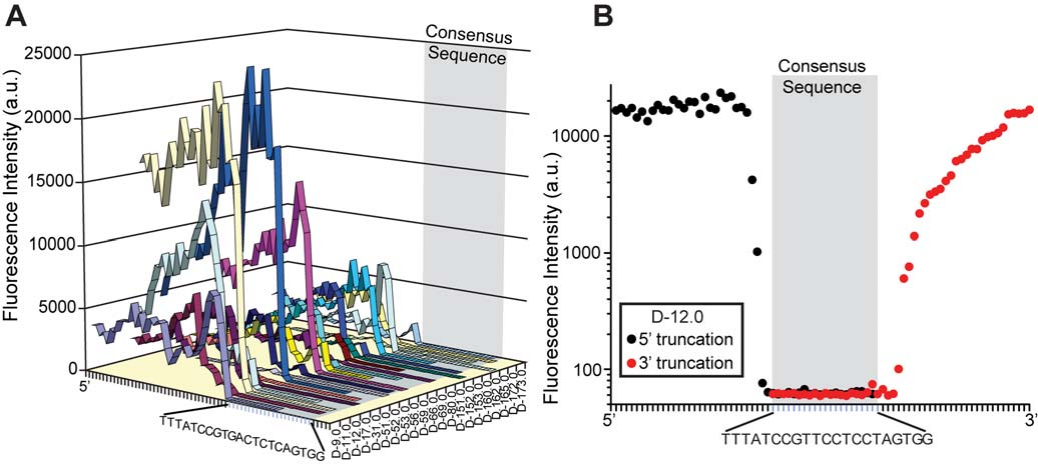
Functional analysis of aptamer truncates elucidates consensus binding sequence. (A) Alignment of 5′ truncation series (10 nM IgE) to the consensus sequence of the parent aptamers. A precipitous drop in IgE binding is observed immediately prior to truncations of the consensus sequence. (B) Data from single 5′ (black circles) and single 3′ (red circles) truncations are superimposed and aligned by consensus sequence of the parent aptamer (clone D-12.0) Loss of IgE binding is evident as the truncations near the consensus sequence for both the 5′ and 3′ ends.

The large amount of sequence dependent binding data made available using high density aptamer arrays provides an opportunity to identify key regions responsible for function. Aligning the consensus sequences of the parent aptamers identifies that the precipitous fluorescence signal drop-off for all 5′ truncates occurs within a few bases of the previously identified consensus sequence. This is illustrated in **Figure 4A** where the consensus sequence of each aptamer has been aligned (highlighted region) and IgE binding deteriorates as the 5′[1] truncation series approaches the consensus sequences. A similar although less dramatic effect was observed for the 3′ truncations and as **Figure 4B** clearly demonstrates, the combination of the 5′ and 3′ truncation array data can be used to identify the consensus binding sequence. These data are consistent with and provide substantial experimental support for previous conclusions drawn from visual analysis of the full-length aptamer sequences and binding data using a single truncate [16]. It is reassuring that the array results are consistent with previous studies and this consistency provides the underpinnings to further exploit this approach for other aptamer sequences to identify important sequence-function relationships.

There are features in the sequence-function data that suggest factors in addition to binding affinity are responsible for the observed fluorescence intensity at any given feature. Most notable is the difference between the 5′ truncation series and 3′ truncation series (**Figure 3A** and **B**, respectively). While decreased affinity associated with 3′ truncations is certainly a possible explanation of the slow decrease in fluorescence intensity across the mutation series, there are additional factors that may contribute to this phenomenon such as the inherent differences in the way the 5′ and 3′ truncations were generated. As depicted in **Figure 1**, the 5′ truncations simply remove a base from the 5′ end of the oligonucleotide while all of the remaining nucleotides stay in the same relative position to the array surface and therefore have similar if not improved accessibility to the IgE in solution. A portion of the increased binding observed with the serial 5′ truncates could be ascribed to greater binding site accessibility as long stretches of nucleotides not involved in target binding are removed. In contrast, the 3′ truncations remove a nucleotide from the base of the aptamer, effectively pulling the consensus sequence closer to the surface and potentially decreasing accessibility to the protein target due to steric crowding. The combined 3′ and 5′ truncates show a balance of these effects with significant fluorescence signals maintained in most cases until the consensus sequence is affected. The true origin of these phenomena will require in-depth investigation regarding the surface effects associated with these high density aptamer arrays. In particular, the results suggest that future chip designs should include a compensatory T insertion for every nucleotide that is removed during truncation. The resulting oligo-T stilts would result in sequences all of the same length, potentially minimizing the putative effects on binding by steric crowding near the array surface. Furthermore, the oligonucleotide density per feature can be modulated to explore the possible correlation between probe density, individual aptamer folding, and target accessibility. These strategies will be incorporated into future experiments with high density aptamer arrays.

### Correlation of fluorescence intensity and binding affinity

As discussed, IgE binding measured by fluorescence intensity at any one feature could be impacted by a number of factors. In addition to oligonucleotide sequence dependent target affinity, surface immobilization affects aptamer binding properties. Although determination of true solution K_d_s using these arrays was not possible (also observed in [15]), relative binding affinity may be determined but only to the extent that surface effects such as binding site availability are neutralized between sequences that are being compared. This is most likely to be the case for aptamers of similar lengths and relative position of consensus binding sites (i.e. they are closely related in the truncation series). Katilius *et al*. recently investigated the relationship between aptamer array-based fluorescence and solution-based dissociation constants, demonstrating that qualitative changes in fluorescence intensity within an array correlate to commensurate differences in binding affinity [15].

### Two-dimensional structure – function relationships

Computational methods exist to model DNA secondary structure which, when combined with sequence dependent binding data, enable predictions of how secondary structure impacts function. The program Mfold [17] was used to calculate the lowest free energy secondary structures for representative aptamers and a series of their cognate truncates. Often for any given sequence several alternative secondary structures are predicted within a relative small range of free energies. The knowledge of functional secondary structures can have a significant impact on optimizing desired properties as well as contribute to building a stronger foundation for aptamer engineering and de novo design of aptamer function. Detailed and extensive data that correlate sequence and structural elements required for binding can be used to refine models and focus on those secondary structures that most likely contribute to target binding.

The secondary structures of the highest intensity truncate from all 21 IgE aptamers were surveyed to identify common structural themes. Interestingly, the secondary structure originally proposed, prior to the availability of the M-fold program, is not predicted as one of lowest energy secondary structures. Rather, the lowest energy structures place the consensus sequence entirely in a loop (**Figure S4**). Analysis of the 21 individual aptamers revealed that 90% of the highest intensity truncates demonstrate a loop that is exclusively comprised of the entire consensus sequence (**Figure S5**). Of these, 74% are the lowest free energy structures. There is greater variability in the base-pairing requirement within the stem. All but one of the highest intensity truncates have at least three base pairs comprising the stem. However, as many as 12 base pairs are found stabilizing the stem structure. While all but one consensus sequence are terminated by a T and G (5′ and 3′ termini, respectively), this potential non-Watson-Crick base pair is not linked to IgE binding.

**Figure 5:**
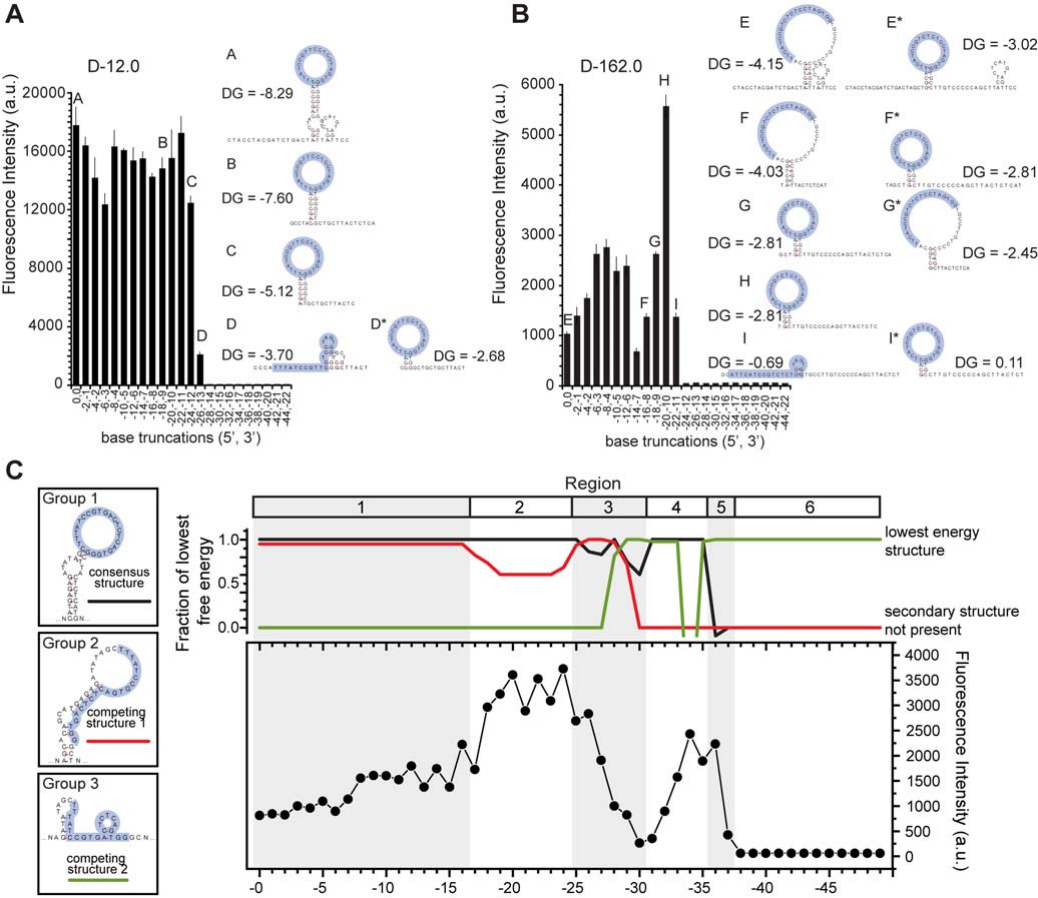
Secondary structure can be correlated to aptamer function. (A,B) Mfold structures of selected truncates are mapped to fluorescence signal intensity (5′[2]3′[1] truncation set). Lowest free energy two-dimensional structures are on the left, and corresponding higher energy structures are denoted by an asterisk (*). (A) Clone D-12.0 demonstrates steady fluorescence signal until the stem stabilizing the loop is perturbed. (B) Clone D-162.0 demonstrates greater variability in fluorescence signal due to two competing structures, of which only one exclusively displays the consensus sequence in the loop. (C) The free energy of observed secondary structures (clone D-66.0, 5′[1]3′[0] truncation set) can be correlated to the observed fluctuations in fluorescence intensity upon IgE binding. Secondary structures predicted by Mfold were consolidated into three groups (left). The corresponding ΔG values of these folding groups are plotted (top) in relation to overall fluorescence intensity observed at each 5′ truncate (bottom). Six unique regions correlating free energy of the competing structural groups with fluorescence intensity are identified (see text for details). All consensus sequences are highlighted. Mfold structures were calculated using experimental parameters (25°C, 137mM NaCl, 1mM MgCl_2_). Error bars are 1 s.d. of triplicate data points and all ΔG values are in kcal/mol.

Two representative aptamers were chosen to correlate secondary structure predictions with actual binding intensities along sequential 5′[2]3′[1] truncations. **Figure 5** maps representative lowest energy Mfold structures onto the aptamer microarray binding data. The truncations of D-12.0 exhibited overall steady fluorescence intensity signal as the truncation progressed (**Figure 5A**), and were characterized by loop structures exclusively displaying the consensus sequence. At a certain threshold, from structure C to D, the stem was sufficiently destabilized such that the lowest free energy structure no longer displayed the consensus sequence in the loop (although a higher energy fold, D*, maintains this structure). The low fluorescence signal observed in structure D was completely abrogated upon a further two base truncation since the consensus stem:loop structure could no longer form. Clone D-162 exhibited drastic shifts in fluorescence intensity signal (**Figure 5B**), due in large part to an alternate lowest energy folding structure in which the loop region was not exclusively comprised of the consensus sequence (E and F). A shift was seen between structures F and G, where the consensus structure became lower energy (G) than the larger, alternate loop structure (G*). The sequence corresponding to structure H, which can only fold into the consensus structure, exhibited the greatest binding intensity. These analyses strongly suggest that exclusive display of the consensus sequence within the loop was required for the highest degree of IgE binding.

While free energy of the lowest free energy structure does not directly correlate to fluorescence intensity, differences in free energy (ΔG) between competing secondary structures track well with IgE binding. This is observed when comparing the ΔG values of the dominant structural species present in the 5′ truncation series of clone D-66.0 (**Figure 5C**). The three groups of competing secondary structures include those folds that place the consensus sequence wholly within the loop (Group 1), alternate folds sequestering the majority of the consensus sequence in the stem (Group 2), and secondary structures of shorter truncates that have little secondary structure (Group 3). The interplay between the ΔG of these three groups and the observed fluorescence intensity profile of the truncation series can be parsed into 6 distinct regions. The free energy of Groups 1 and 2 are similar and remain constant in region 1, and any increases in fluorescence intensity may be due to increased accessibility of the consensus sequence by the removal of 5′ regions not involved in IgE binding until the truncations disrupt the stem structure of Group 2. At this point (region 2), the increase in fluorescence intensity is attributed to a higher propensity for the correct consensus fold (Group 1) to occur, hence increasing IgE binding. Upon further 5′ truncations, however, the ΔG of Group 1 decreased to that of Group 2 due to stem disruption and Group 3 structures are introduced (region 3). The ability to fold into three thermodynamically similar structures reduced the efficacy of aptamer binding to IgE, resulting in a dramatic decrease in intensity. In region 4, further 5′ truncations completely abrogated Group 2, effectively decreasing the competition for alternative structures and resulted in an increase in IgE binding which continued into region 5. It is worth noting that less than two bases comprise the stem of Group 1 structures in region 5 and it is possible that binding-induced folding may be an operative binding mechanism in this region. The ability of target binding to induce folding of an unstructured or weakly folded aptamer is well documented, and is the basis for several aptamer-based detection platforms [18]–[20]. Lastly, as the 5′ truncations begin removing recognition bases of the consensus sequence, the Group 1 structure is no longer favorable or even possible and no IgE binding was observed (region 6). This analysis extends the observations made in **Figures 5A** and **5B**, supporting the notion that overall aptamer binding is dictated by a complex set of interactions including target accessibility to the binding sequence, thermodynamic stability of competing secondary structures, and target-induced changes in kinetics of aptamer folding.

In conclusion, high density microarray technology was used to create thousands of aptamer sequence permutations on a single array and perform thousands of experiments in parallel. While this is only one set of experiments against a single protein target it bodes well for the generality of applying high density aptamer arrays against other protein targets. The custom arrays are essentially available on demand from commercial vendors, such that the process of optimizing aptamers for a particular function can be greatly accelerated and can be done across a large number of starting aptamers sequences. This approach promises to relieve a significant bottleneck that exists in optimizing aptamers where current approaches limit evaluation to just a handful of sequences. This is especially true for selections that identify disparate sequences lacking clear consensus regions. In these cases, the lack of a clear starting point would require initial hit-or-miss identification of the binding domain before optimization can proceed. This approach can also be used to initially screen thousands of clones from a single SELEX experiment, rather than the 20–50 clones typically analyzed, to providing an extremely thorough interrogation of potential high affinity aptamers. Furthermore, the high throughput achievable with the aptamer arrays should allow rapid assessment of aptamer specificities. In addition, future aptamer array applications may incorporate optimization of additional properties such as binding induced signaling for sensing applications. This could include using reporter fluorescent molecules such as FRET pairs that change signal upon binding or electrode arrays in which target binding induced changes in electrochemical reactions are used to generate a signal. Indeed, the Combimatrix electrode array microarray format has the potential to be used much like the Agilent arrays were used here but with an electronic readout. In addition to practical applications, high density aptamer arrays will likely have an impact on our basic understanding of the biophysics of these molecules. The large sets of sequence-function data provide a foundation from which to build our understanding of aptamer structure-function relationships and ultimately improve our ability to engineer and design this class of high affinity reagent.

## Materials and Methods

### Materials

Bovine serum albumin (BSA), casein, dextran sulfate (DS) and Tween-20 (T-20) were obtained from Sigma-Aldrich (St. Louis, MO). Human myeloma Immunoglobulin E was purchased from Athens Research and Technology (Athens, GA). Cy3-labeled landing-lights DNA (LL-DNA) required for visualization of array fiducials was HPLC purified (Integrated DNA technologies, Coralville, IA) and used as received: 5′ CCAGTGACTTTCGTCACTGGAAAACGATCGTTTCCGATCGAAAAGCTAGCTTTCGCTAGC/3Cy3Sp/3′. All other reagents were obtained from Sigma-Aldrich.

### Array design/synthesis

Aptamer arrays were purchased from Agilent Technologies. Each slide consisted of 8 identical subarrays of *ca.* 15,000 features. Features were randomly oriented in triplicate within each subarray. Full length IgE aptamer clones from Wiegand *et al.* [16] (see **Table S1** for sequence information) were serially truncated in the following fashion:

5′[1]3′[0] = single base 5′ truncations

5′[0]3′[1] = single base 3′ truncations

5′[1]3′[1] = single base 5′ and 3′ truncations

5′[1]3′[2] = single base 5′ truncations and two base 3′ truncations

5′[2]3′[1] = two base 5′ truncations and single base 3′ truncations

Each sequence on the array was synthesized with a 3′ T_10_ spacer. Serial truncations of a PDGF aptamer [8] (clone A36) were included as negative controls.

### IgE labeling

IgE was fluorescently labeled with the DyLight 549 according to manufacturer's instructions (Pierce Biotechnology, Rockford, IL). Briefly, IgE (1 mg/mL) was incubated for 1 hour with the DyLight 549 ester (15 mM potassium phosphate, 150 mM NaCl, 50 mM borate, pH 7.4). Free dye was removed using the supplied purification resin and dialysis with IgE binding buffer (8.1 mM N_2_HPO_4_, 1.1 mM KH_2_PO_4_, 2.7 mM KCl, 137 mM NaCl, 1 mM MgCl_2_, 138 mM NaCl, pH 7.4). Dye:IgE ratio was determined to be 5.1. Protein was aliquoted and stored at −20°C.

### Binding experiments

Slides were assembled with 8-well gaskets in an Agilent hybridization chamber for blocking and sample incubations. Each gasket was filled with 65 µL of blocking or sample solutions. Initial screening of incubation parameters tested the effects of different combinations of BSA (10 and 50 mg/mL), dextran sulfate (0.2 mg/mL), casein (10 mg/mL) and Tween-20 (0.1% v/v) on signal and background intensities. Casein and Tween-20 were identified as important constituents for optimal signal:noise ratios (see **Figure S1**), so slides were henceforth blocked 1 hr at room temperature with blocking buffer (10 mg/mL casein, 0.1% T-20 (v/v), 8.1 mM N_2_HPO_4_, 1.1 mM KH_2_PO_4_, 2.7 mM KCl, 137 mM NaCl, 1 mM MgCl_2_, 138 mM NaCl, pH 7.4). Slides were disassembled in binding buffer and rinsed a further 5 minutes at room temperature. Labeled IgE was diluted in blocking buffer supplemented with 0.2 nM LL-DNA and incubated with the arrays for 2 hours at room temperature. Arrays were again disassembled in binding buffer and rinsed 5 times for 3 minutes in >50 mL binding buffer. In early experiments, slides were washed with binding buffer containing 0.1% Tween-20., though subsequent optimization determined this was unnecessary. Slides were placed in empty 50 mL conical tubes and dried by centrifugation for 5 minutes at 2000× *g*. Slide were purged with nitrogen and scanned within 16 hours.

### Data analysis

Slides were scanned using the Agilent DNA microarray scanner at a 5 micron resolution. In cases of saturation, the slides were scanned using the extended dynamic range (XDR) feature. Features were extracted using Agilent's Feature Extraction Software and compiled into a database for subsequent analysis. Oligonucleotide secondary structures were predicted using Mfold [17], setting folding parameters to the experimental conditions (137 mM Na^+^, 1 mM Mg^++^, 25°C).

## Supporting Information

Table S1 (0.04 MB DOC)

Figure S1 Signal-to-noise optimization of aptamer array. (A) The blocking effects of (1–3) bovine serum albumin (BSA), (4–6) casein, and (3,6) dextran sulfate (DS) were tested during the 1 hr pre-block step prior to IgE addition. Further, incubation of IgE in the presence of BSA or casein was examined. (B) Representative regions of subarrays correlated to blocking conditions. All conditions resulted in features that were uniform in size and shape. However, BSA caused non-uniformity in intensity distribution within individual features, resulting in a higher signal intensity at the feature periphery. Subarrays blocked with casein displayed uniform intensity throughout all features. (C) Signal to noise ratios were obtained by comparing fluorescence intensities of full length D-12.0 with full length PDGF (negative control). Error bars represent 1 s.d. of triplicate data points.
(1.57 MB TIF)

Figure S2 Subarray correlation and variability coefficient analysis. (A,B) The average of triplicate data points were used in the correlation analyses of (A) intra- and (B) interslide subarrays. (C,D) Variability coefficient analyses of intra- and interslide subarrays, respectively. Mean coefficient of variability values for intra- and interslide arrays were 0.0576 and 0.0686, respectively. Since the two slides used for the inter-slide subarray comparisons were not washed identically, the Slide1 Array1–3 data were multiplied by a correction factor of 1.8417 (the slope of the correlation plot in B, see Figure 2) to normalize the fluorescence intensity values (see manuscript text). However, this correction factor does not take into consideration the non-linear relationship between background (shaded area in B and D) and sample fluorescence intensities. For this reason, all data points with normalized mean fluorescence intensities below 130 a.u. were not used in determination of the inter-slide average coefficient of variability.
(1.91 MB TIF)

Figure S3 Fluorescence signal intensity is dependent on protein concentration. Identical subarrays on a single microarray slide were incubated with increasing concentrations of labeled IgE. Four truncates of clone D-12.0 (inset) were analyzed, demonstrating a systematic increase in fluorescence intensity at higher concentrations of IgE. While no plateau in intensity was observed, the traces for the individual clones can be correlated to fluorescence intensity at a single IgE concentration (inset).
(0.53 MB TIF)

Figure S4 Comparison of original proposed secondary structures with Mfold lowest free energy structures. (A) Wiegand et al. originally proposed a stem:loop structure (left) in which the first 5′ T residue of the consensus sequence was bulged and not paired. Base pairs required for stem formation beyond the consensus sequence are denoted by N-N'. Using Mfold, which was not available to Wiegand et al., the predicted consensus structure identified in 90% of the clones analyzed on the microarray placed the entire consensus sequence within the unstructured loop (right). In 40% of these, the 5′ T is paired to the 3′ G of the consensus sequence. (B) The differences in free energy were compared between the secondary structure proposed by Wiegand et al. (left) and the Mfold predicted consensus structure (right). Folding constraints were required to achieve the folding on the left, whereas the structure on the right is the default, unconstrained lowest energy fold. Without constraints, the structure on the left is not observed in the 46 folds identified within 90% of the optimal fold ΔG.
(0.46 MB TIF)

Figure S5 Mfold structures and ΔG calculations of the highest intensity truncates for all 21 clones. Consensus sequences are highlighted in blue. Yellow shading represents sequences that are not characterized by a loop completely comprised of the consensus sequence. Green shading represents structures for which folding constraints were required to remove any secondary structure within the consensus loop (D-59.0) or to place the entire consensus sequence within the loop (D-31.0). All clones truncates are in order of highest to lowest fluorescence intensity, as indicated by graph at bottom right (error bars represent 1 s.d. from triplicate samples).
(1.85 MB TIF)
