# Supplementary material for: Massively Parallel Interrogation of Aptamer Sequence, Structure and Function
Source: PLoS One. 2008 Jul 16;3(7):e2720. doi: 10.1371/journal.pone.0002720 (PMC2444025; doi:10.1371/journal.pone.0002720)
Supplement: Table S1 — (0.04 MB DOC) [file pone.0002720.s001.doc]

**Massively Parallel Interrogation of Aptamer Sequence,**

**Structure and Function**

Nicholas O. Fischer^1^, Jeffrey B.-H. Tok^1^, Theodore M. Tarasow^2^,*

^1^Chemistry, Materials, Earth and Life Sciences Directorate, Lawrence Livermore National Laboratory, 7000 East Ave., Livermore, CA 94550. ^2^Tethys Bioscience, Inc., 5858 Horton Street #550, Emeryville, CA 94608

**Table S1.** Sequences of full length aptamers utilized in study.

| **IgE aptamers** | | |
| --- | --- | --- |
| D 9.0 | | CTACCTACGATCTGACTAGCATGGGAGAACACTTAGCCTTCATCCGTTCCTCCTAGTGGGGCTTACTCTCATGTAGTTCC |
| D 11.0 | | CTACCTACGATCTGACTAGCACCGGAGTACTTCATCCGTCCCTTCTAGTGGGTACCCGTAGCTTACTCTCATGTAGTTCC |
| D 12.0 | | CTACCTACGATCTGACTAGCCTACAGCCCATTTATCCGTTCCTCCTAGTGGTGGGCTGCTGCTTACTCTCATGTAGTTCC |
| D 17.0 | | CTACCTACGATCTGACTAGCCAATGAGTGTACCACGTTTATCCGTCCCTCCTAGTGGCGTGCTTACTCTCATGTAGTTCC |
| D 31.0 | | CTACCTACGATCTGACTAGCACGGGTGAGTTGATCCGTCACTCTTAGTGGTGAACCTTGTGCTTACTCTCATGTAGTTCC |
| D 51.0 | | CTACCTACGATCTGACTAGCAAGGCGACTACTTTATCCGTTTCTCTTAGTGGGTATCCGGCTTACTCTCATGTAGTTCC |
| D 52.0 | | CTACCTACGATCTGACTAGCCGCGCGTACGAGCACCTTCATCCGTCCCTCCTAGTGGGGTGCTTACTCTCATGTAGTTCC |
| D 53.0 | | CTACCTACGATCTGACTAGCCGAAGTTAATTTATCCGTCCCTCCTAGTGGTCTTAACAGCGGCTTACTCTCATGTAGTTCC |
| D 56.0 | | CTACCTACGATCTGACTAGCTGCTGGACAATTGATCCGTTACTCTTAGTGGTTGTGTGCTGCTTACTCTCATGTAGTTCC |
| D 59.0 | | CTACCTACGATCTGACTAGCGATGGGAGCTTTATCCGTTCACTCTCAGTGGGCTCCTCAGGCTTACTCTCATGTAGTTCC |
| D 66.0 | | CTACCTACGATCTGACTAGCACAGCATGAGAGATATAGCTTTATCCGTGACTCTCAGTGGGCTTACTCTCATGTAGTTCC |
| D 69.0 | | CTACCTACGATCTGACTAGCGGCCGTAAGCAACCTTTATCCGTAATCTCTCAGTGGGGTAGCTTACTCTCATGTAGTTCC |
| D 80.0 | | CTACCTACGATCTGACTAGCGTAGCGCGCTTTATCCGTTTCTCCCAGTGGGCGGCGTTCGCTTACTCTCATGTAGTTCC |
| D 151.0 | | CTACCTACGATCTGACTAGCTACCCGCGATGAGAGTAAGTTTATCCGTGTACTCTTAGTGGCTTACTCTCATGTAGTTCC |
| D 152.0 | | CTACCTACGATCTGACTAGCAGGGATGTTCATCCGTTCCTCTCAGTGGCATCCCGTGGCTGCTTACTCTCATGTAGTTCC |
| D 153.0 | | CTACCTACGATCTGACTAGCCAAAGTTAATTTATCCGTCCCTCTCAGTGGTTAACAGCGGCTTACTCTCATGTAGTTCC |
| D 160.0 | | CTACCTACGATCTGACTAGCCAATTGCTGAAGGAAGCATTTATCCGTTCCTCTTAGTGGTGCTTACTCTCATGTAGTTCC |
| D 162.0 | | CTACCTACGATCTGACTAGCTGGCATTCATCCGTCTCTCCTAGTGGTGCCTTGTCCCCCAGCTTACTCTCATGTAGTTCC |
| D 165.0 | | CTACCTACGATCTGACTAGCGTGTGCGGGATCTTTATCCGTTACTCTTAGTGGGTCTCGGCTTACTCTCATGTAGTTCC |
| D 172.0 | | CTACCTACGATCTGACTAGCCTTGCTCCATTTATCCGTTTCTCCCAGTGGTGGTTGCATGGCTTACTCTCATGTAGTTCC |
| D 173.0 | | CTACCTACGATCTGACTAGCAATGGTCCAGCTTTATCCGTCTCTTTCAGTGGGCGTCATTGCTTACTCTCATGTAGTTCC |
|  | |  |
| **PDGF aptamer** | | |
| A36 | GGGATCCGCCTGATTAGCGATACTCCACAGGCTACGGCACGTAGAGCATCACCATGATCCTGTGACTTGAGCAAAATCACCTGCAGGGG | |
