# Supplementary figures and images for: Massively Parallel Interrogation of Aptamer Sequence, Structure and Function
Source: PLoS One. 2008 Jul 16;3(7):e2720. doi: 10.1371/journal.pone.0002720 (PMC2444025; doi:10.1371/journal.pone.0002720)

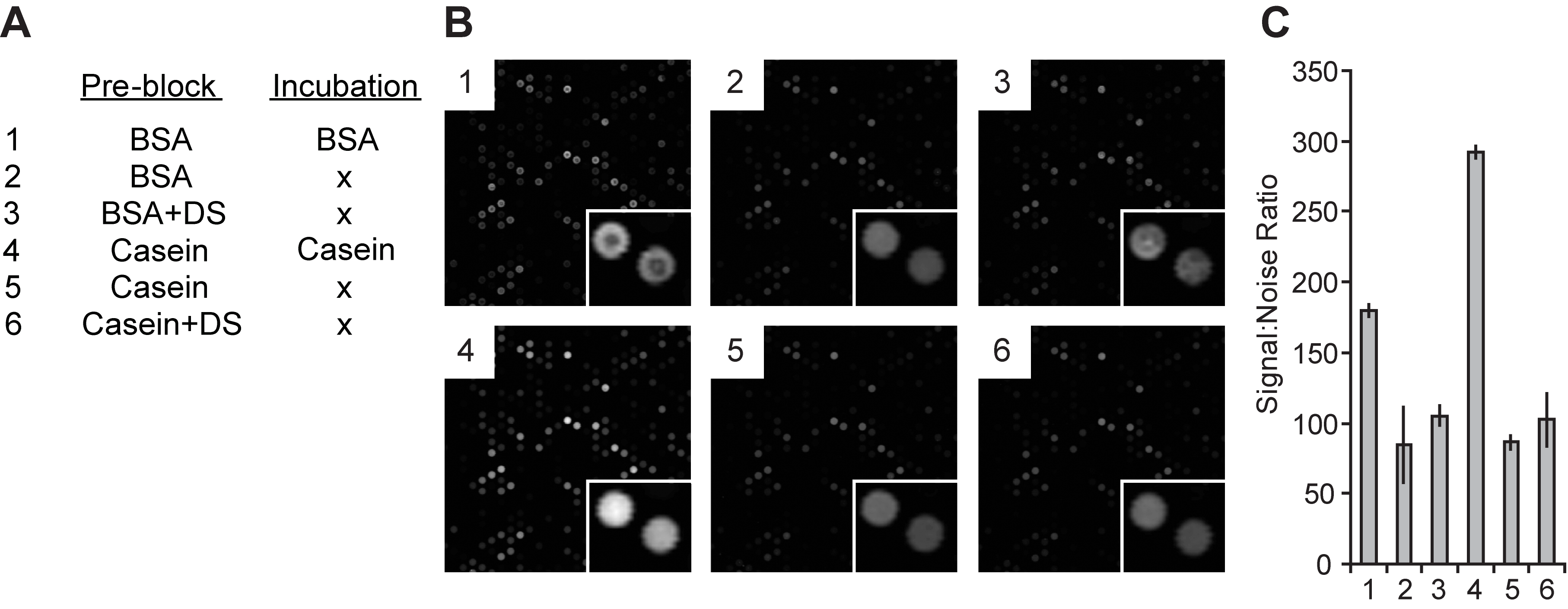

Supplement: Figure S1 — Signal-to-noise optimization of aptamer array. (A) The blocking effects of (1–3) bovine serum albumin (BSA), (4–6) casein, and (3,6) dextran sulfate (DS) were tested during the 1 hr pre-block step prior to IgE addition. Further, incubation of IgE in the presence of BSA or casein was examined. (B) Representative regions of subarrays correlated to blocking conditions. All conditions resulted in features that were uniform in size and shape. However, BSA caused non-uniformity in intensity distribution within individual features, resulting in a higher signal intensity at the feature periphery. Subarrays blocked with casein displayed uniform intensity throughout all features. (C) Signal to noise ratios were obtained by comparing fluorescence intensities of full length D-12.0 with full length PDGF (negative control). Error bars represent 1 s.d. of triplicate data points. (1.57 MB TIF) [file pone.0002720.s002.tif]

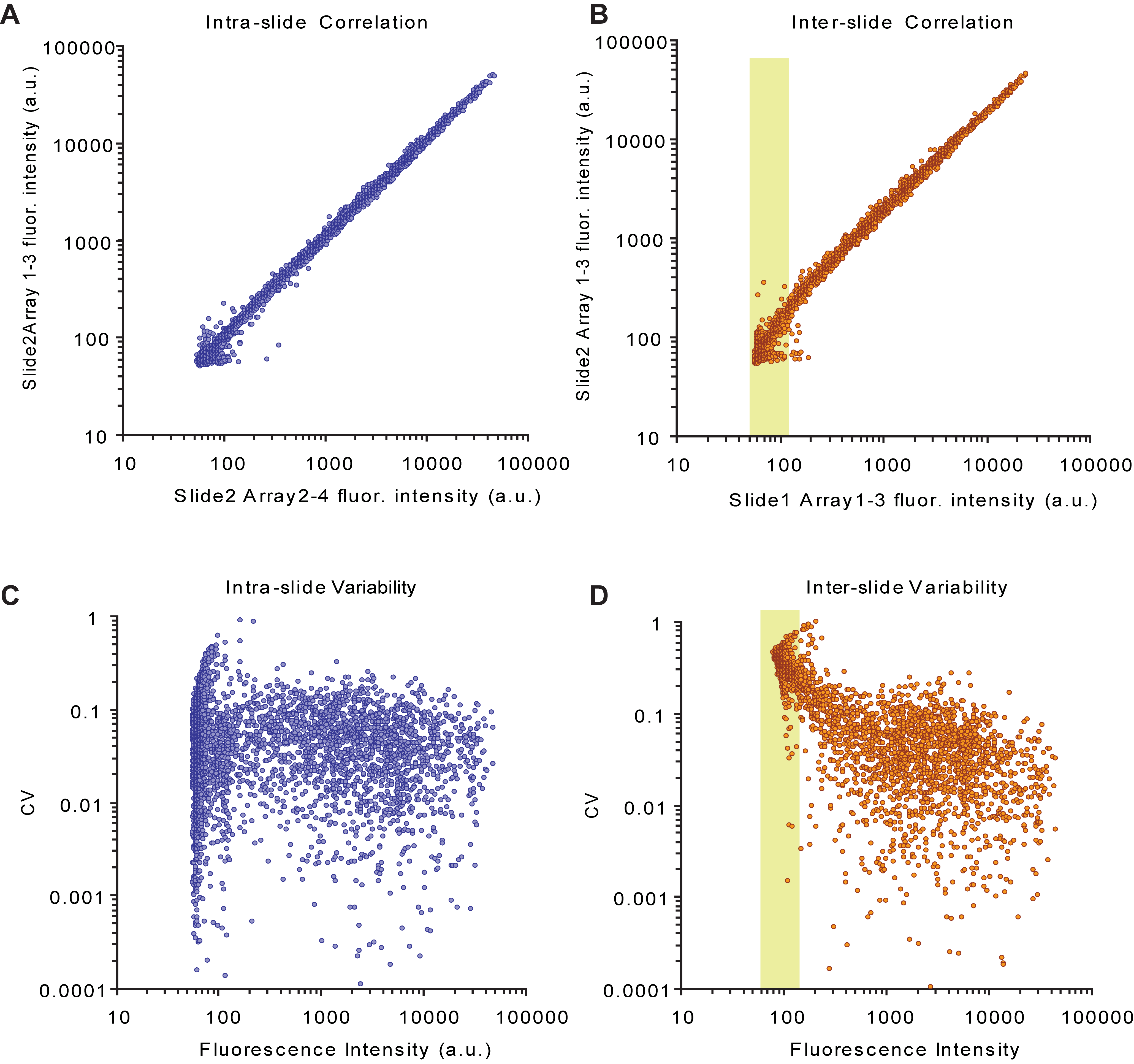

Supplement: Figure S2 — Subarray correlation and variability coefficient analysis. (A,B) The average of triplicate data points were used in the correlation analyses of (A) intra- and (B) interslide subarrays. (C,D) Variability coefficient analyses of intra- and interslide subarrays, respectively. Mean coefficient of variability values for intra- and interslide arrays were 0.0576 and 0.0686, respectively. Since the two slides used for the inter-slide subarray comparisons were not washed identically, the Slide1 Array1–3 data were multiplied by a correction factor of 1.8417 (the slope of the correlation plot in B, see Figure 2) to normalize the fluorescence intensity values (see manuscript text). However, this correction factor does not take into consideration the non-linear relationship between background (shaded area in B and D) and sample fluorescence intensities. For this reason, all data points with normalized mean fluorescence intensities below 130 a.u. were not used in determination of the inter-slide average coefficient of variability. (1.91 MB TIF) [file pone.0002720.s003.tif]

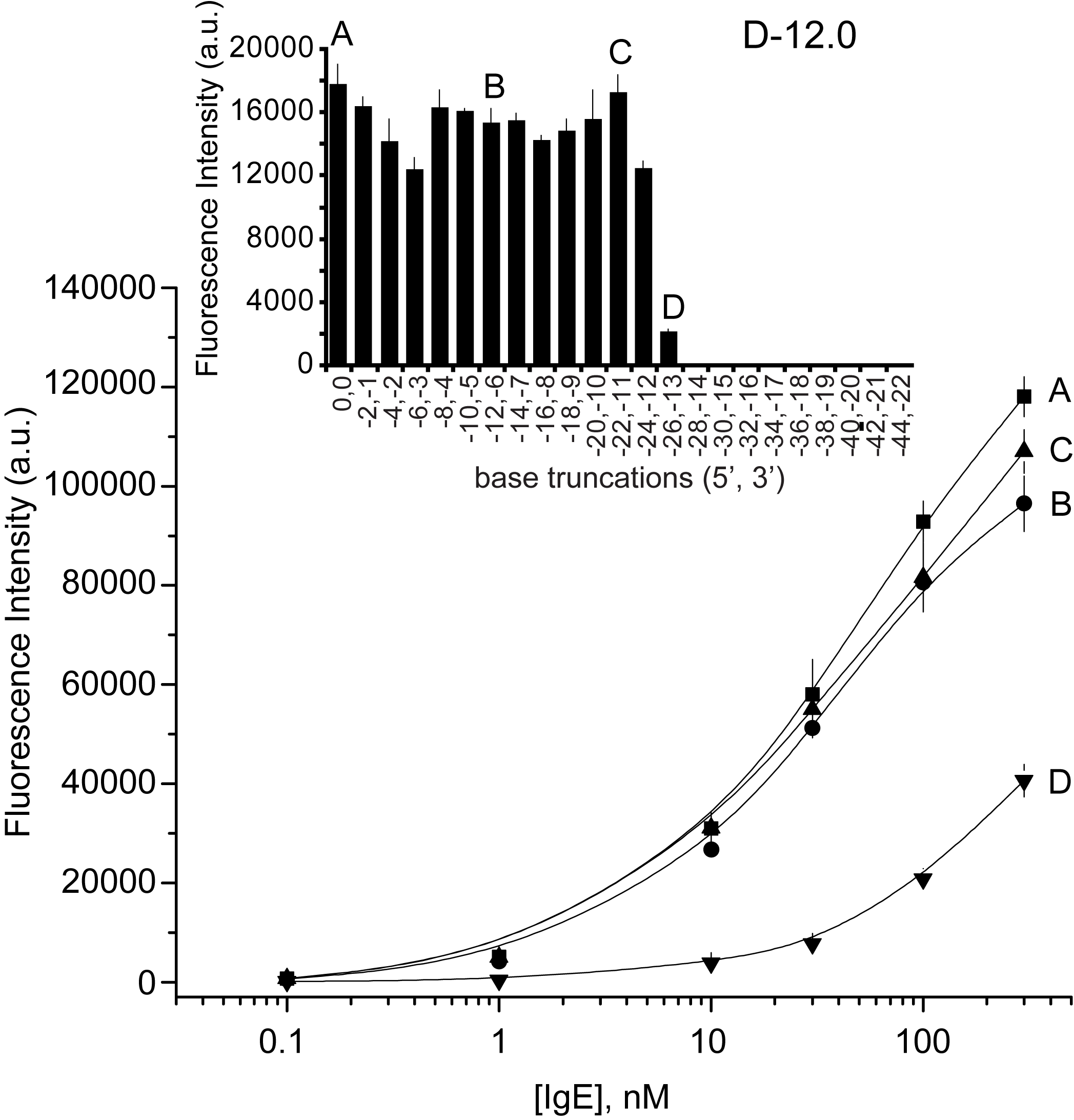

Supplement: Figure S3 — Fluorescence signal intensity is dependent on protein concentration. Identical subarrays on a single microarray slide were incubated with increasing concentrations of labeled IgE. Four truncates of clone D-12.0 (inset) were analyzed, demonstrating a systematic increase in fluorescence intensity at higher concentrations of IgE. While no plateau in intensity was observed, the traces for the individual clones can be correlated to fluorescence intensity at a single IgE concentration (inset). (0.53 MB TIF) [file pone.0002720.s004.tif]

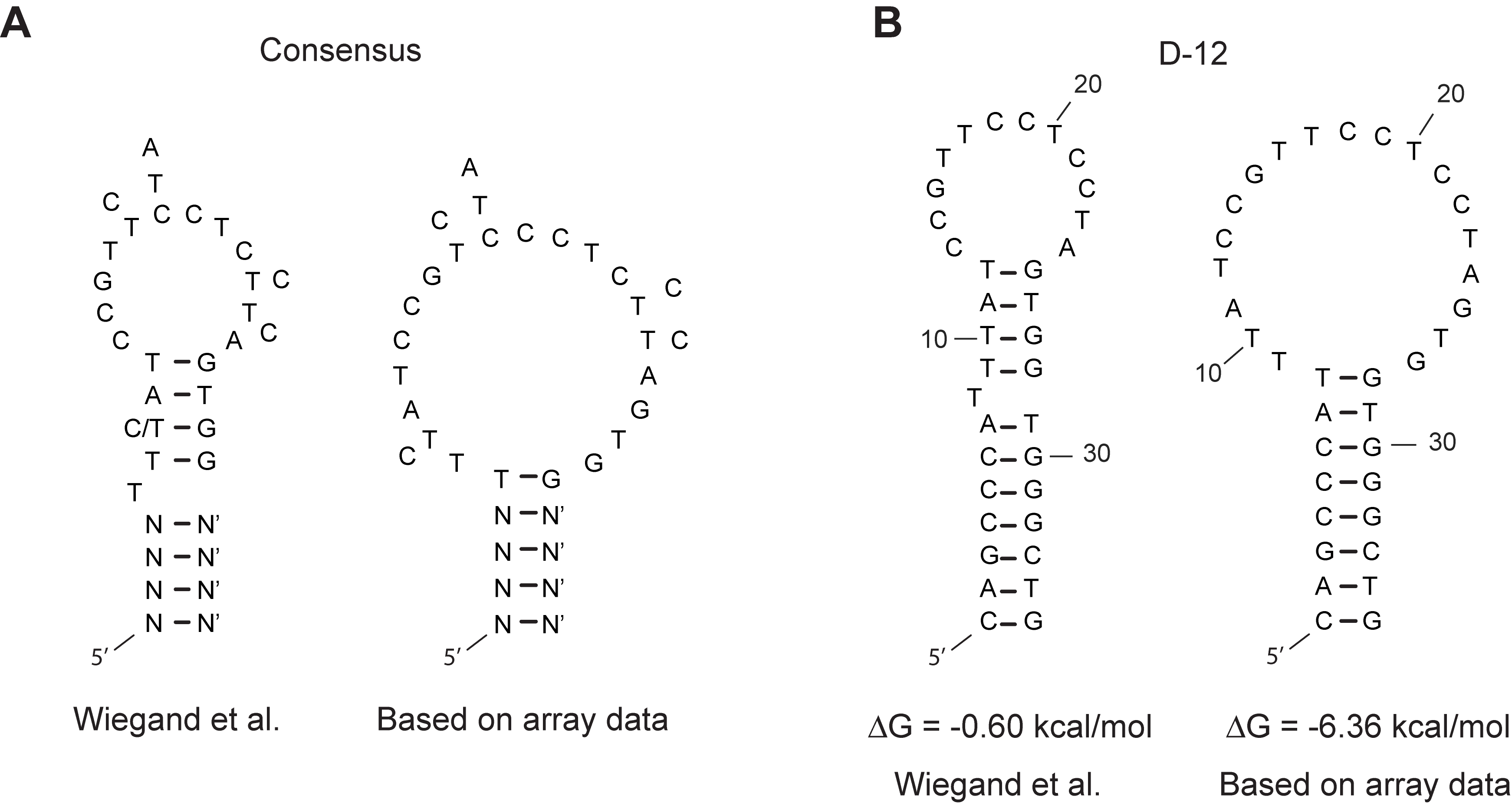

Supplement: Figure S4 — Comparison of original proposed secondary structures with Mfold lowest free energy structures. (A) Wiegand et al. originally proposed a stem:loop structure (left) in which the first 5′ T residue of the consensus sequence was bulged and not paired. Base pairs required for stem formation beyond the consensus sequence are denoted by N-N'. Using Mfold, which was not available to Wiegand et al., the predicted consensus structure identified in 90% of the clones analyzed on the microarray placed the entire consensus sequence within the unstructured loop (right). In 40% of these, the 5′ T is paired to the 3′ G of the consensus sequence. (B) The differences in free energy were compared between the secondary structure proposed by Wiegand et al. (left) and the Mfold predicted consensus structure (right). Folding constraints were required to achieve the folding on the left, whereas the structure on the right is the default, unconstrained lowest energy fold. Without constraints, the structure on the left is not observed in the 46 folds identified within 90% of the optimal fold ΔG. (0.46 MB TIF) [file pone.0002720.s005.tif]

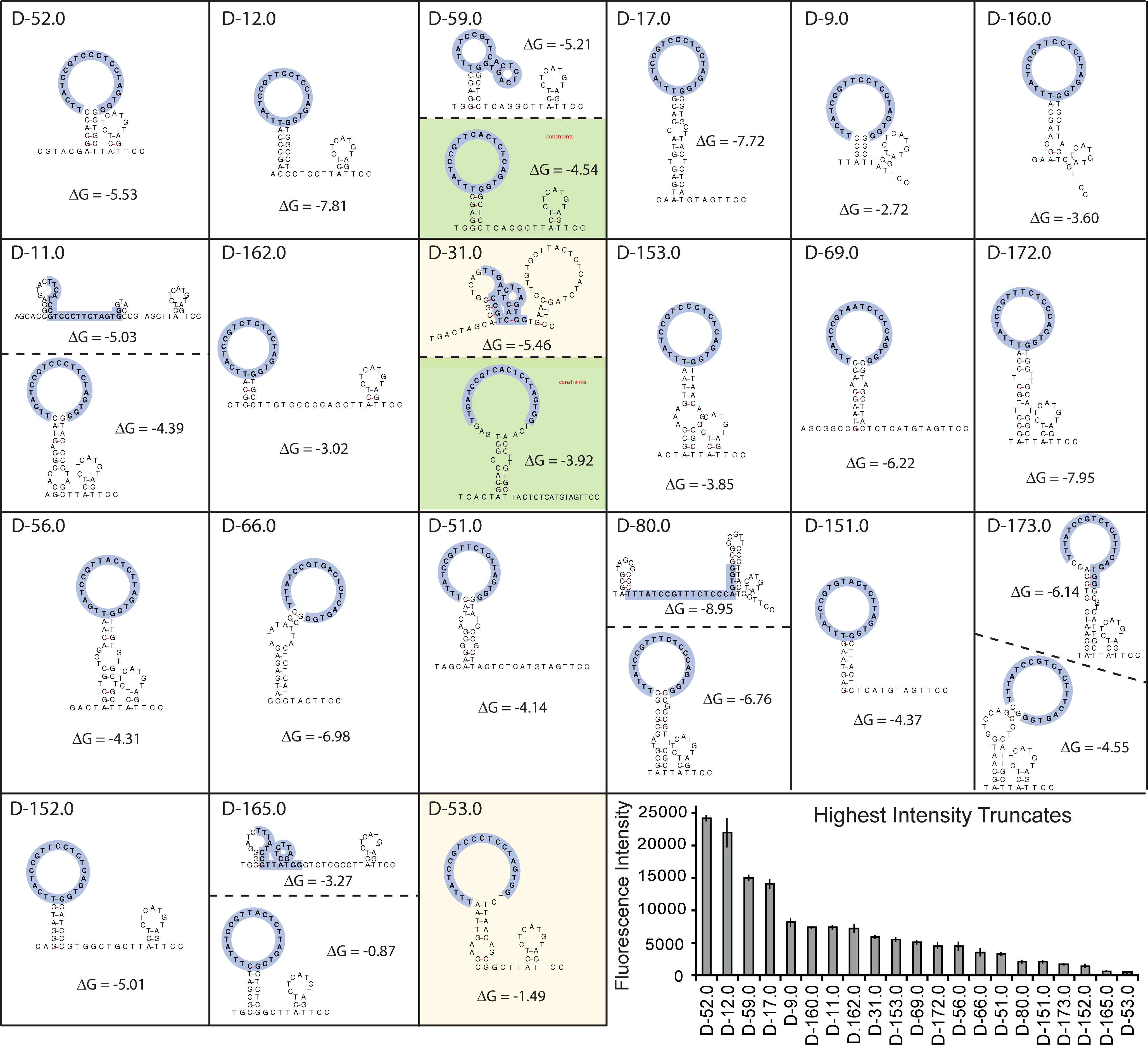

Supplement: Figure S5 — Mfold structures and ΔG calculations of the highest intensity truncates for all 21 clones. Consensus sequences are highlighted in blue. Yellow shading represents sequences that are not characterized by a loop completely comprised of the consensus sequence. Green shading represents structures for which folding constraints were required to remove any secondary structure within the consensus loop (D-59.0) or to place the entire consensus sequence within the loop (D-31.0). All clones truncates are in order of highest to lowest fluorescence intensity, as indicated by graph at bottom right (error bars represent 1 s.d. from triplicate samples). (1.85 MB TIF) [file pone.0002720.s006.tif]
